# Biomimetic vascular tissue engineering by decellularized scaffold and concurrent cyclic tensile and shear stresses

**DOI:** 10.1007/s10856-023-06716-4

**Published:** 2023-03-14

**Authors:** Hamed Omid, Sorosh Abdollahi, Shahin Bonakdar, Nooshin Haghighipour, Mohammad Ali Shokrgozar, Javad Mohammadi

**Affiliations:** 1 Department of Life Science Engineering, Faculty of New Sciences and Technologies, University of Tehran, Tehran, Iran; 2 National Cell Bank of Iran, Pasteur Institute of Iran, No. 69, Pasteur Ave, Tehran, 1316943551 Iran; 3 School of Metallurgy and Materials Engineering, Iran University of Science and Technology, Tehran, Iran

## Abstract

Decellularization by chemical approaches has harmful effects on extracellular matrix (ECM) proteins, and damages lots of functional peptides and biomolecules present in the ultrastructure. In this study, we employed a combination of chemical and physical decellularization methods to overcome these disadvantages. The induced osmotic pressure by hypertonic/hypotonic solutions dissociated and removed most of cellular membranes significantly without any detergent or chemical agent. In total, 0.025% trypsin solution was found adequate to remove the remaining debrides, and ultimately 1% Triton X-100 was utilized for final cleansing. In addition, conducting all the decellularization processes at 4 °C yielded an ECM with least damages in the ultrastructure which could be inferred by close mechanical strength and swelling ratio to the native vessel, and high quality and quantity of cell attachment, migration and proliferation which were examined by optical microscopy and scanning electron microscopy (SEM) of the histology samples. Moreover, the obtained biological scaffold (BS) had no cytotoxicity according to the MTT assay, and this scaffold is storable at −20 °C. Employing bioreactor for concurrent cyclic tensile and shear stresses improved the cell migration into pores of the BS and made the cells and the scaffold compact in analogous to native tissue. As opening angle test showed by decellularizing of the blood vessel, the residual stress dropped significantly which revealed the role of cells in the amount of induced stress in the structure. However, intact and healthy ECM explicitly recovered upon recellularization and beat the initial residual stress of the native tissue. The tensile test of the blood vessels in longitudinal and radial directions revealed orthotropic behavior which can be explained by collagen fibers direction in the ECM. Furthermore, by the three regions of the stress–strain curve can be elucidated the roles of cells, elastin and collagen fibers in mechanical behavior of the vascular tissues.

**Graphical Abstract:**

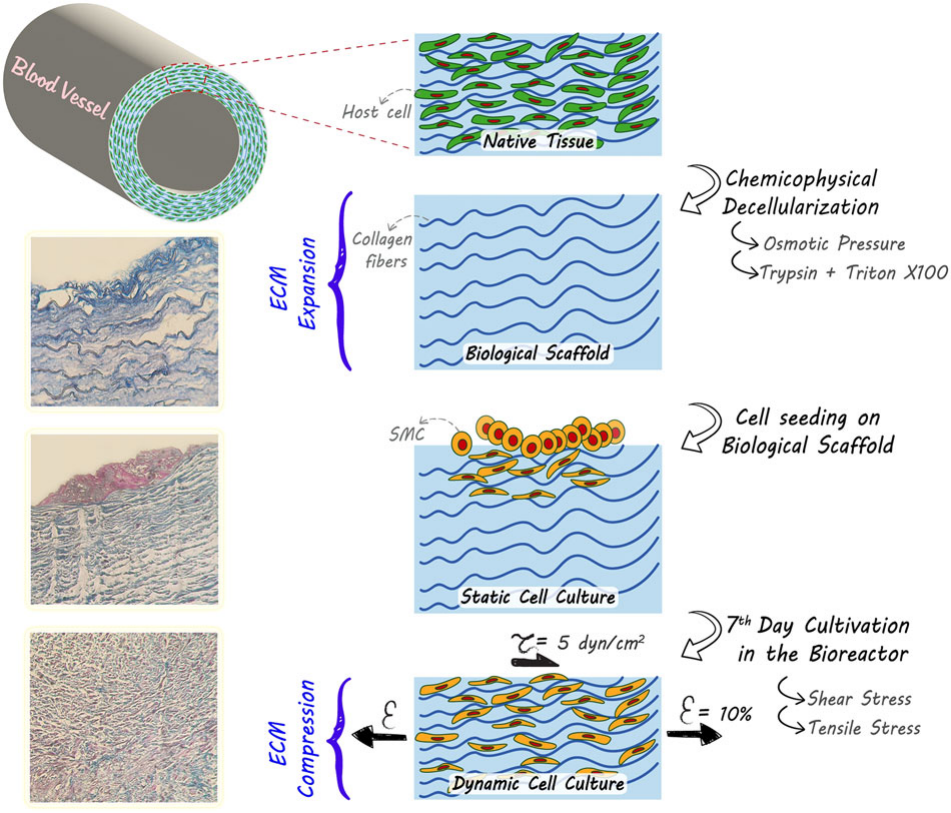

## Introduction

The market for cardiovascular biomaterials and implants is booming due to a rising number of people suffering from vascular diseases [1]; hence, research on synthetic vascular implants is ongoing. However, for advanced cardiovascular diseases, artery bypass graft surgery (taking a blood vessel from another part of the body and implanting it to the damaged artery site [2]) remains the primary therapy, because any of the current methods for fabricating synthetic vascular grafts have not beaten sufficient efficacy to create small-caliber arteries [3, 4]. In addition to dimensions, the biocompatibility of the implant and its capability to be integrated with the host tissue are of essence, otherwise an acute inflammation could be orchestrated causing the rejection of implant [5, 6]. Therein, the concept of tissue regeneration emerges, that a biomimetic 3D microenvironment eases the infiltration of cells, their attachment, and proliferation culturing an engineered organ.

Tissue engineering of vascular grafts was attempted for the first time in 1950s using acellular synthetic polymers to replace occluded arteries [7]; however, they were not up to for the biomimicry of a native three-layered vessel which is composed of endothelial cells, smooth muscle cells (SMCs), and fibroblasts, all attached and organized in ECM [8, 9]. Afterward, a new alternative emerged based on the development of engineered vascular conduits upon the growth of ECs and SMCs within the context of scaffolds with a microstructure analogous to ECM. However, moderated mechanical strength restrained their applicability. On the other hand, the microenvironment provided by these conduits never lead the cells to exhibit regular functionality, in points of either proliferation or protein translation [10, 11].

The insight to employ decellularization-derived vascular grafts promises a range of applications and several advantages in contrast to synthetic materials [12–14]. An adequate decellularization protocol could yield an acellular architecture which is inspired of native organ; therefor the engineered tissue inherits native microvasculature and microstructure [15]. The microvascular network could facilitate the transportation of oxygen and nutrients, and further paves the way for cellular waste removal [16, 17]. On the other hand, the mechanical support that would be provided is remarkable and hosting the cells to demonstrate their habitual behavior and functionality [18, 19]. A huge significance of decellularized grafts in contrast to other synthetic tissues is the preservation of natural signaling pathways that could orchestrate and coordinate the interactions of cells with together and ultimately elevate recellularization [20, 21]. Signaling agents include genes, small molecules, mRNAs, cytokines, and growth factors; upon a less damaging decellularization procedure, many of these pathways could be preserved from the native organ [18].

Though, a full decellularization will never be acquired and always a proportion of cellular components remain, it is absolute necessary to find out an optimized protocol maximizing cell removal, otherwise the recruitment of macrophages and the outbreak of immune rejection will be inevitable. Further, all steps of the processing should be detailed and implemented somehow not to harm the ultrastructure, mechanical properties, and to preserve the signaling pathways as much as possible [22, 23]. Any decellularization protocol requires post-treatment complements, in points of either washing out the detached cells or sterilization [24, 25]. There are two major approaches—chemical and physical—that could be the basis of decellularization [26, 27]; however, for the chemical one, post-treatments should be elaborated carefully to wash out all chemicals and detergent as well, otherwise they would hinder cell attachment and proliferation during recellularization, whereas physical decellularizing treatments do not face this challenge.

We report here the results of a decellularization of vascular engrafts harvested from ovine coronary arteries. The decellularization protocol was employed in a way to having minimal chemical interferences and be mainly physical based employing hypertonic/hypotonic solutions. In the washing cycle with hypertonic saline solution, DNAs are dissociated from proteinaceous membranes. Later, in rinsing with hypotonic solutions an osmotic pressure is generated lysing the cells out of ECM without any detrimental effects on the ultrastructure [28, 29]. Performing all the processing at 4 °C minimized damages to the ECM and kept it fresh. The investigation of physical properties, histological studies, and microscopic visualizations revealed the minimal effect of decellularization on the native architecture of ECM. Moreover, with an in vitro MTT assay the decellularized tissue was inspected and found non-cytotoxic. Ultimately, the resultant engraft was subjected for recellularization with SMCs in a dynamic bioreactor, and the infiltration, attachment, and proliferation of cells was studied.

## Materials and methods

### Harvesting of blood vessels

Ovine coronary arteries were isolated freshly from a slaughter house, under the supervision and ethical board of the faculty of veterinary medicine, University of Tehran. The excessive parts were cut and the remaining tissues were washed twice to remove blood clots, and stored at 4 °C until decellularization.

### Decellularization method

The excess connective tissues on the outer part of adventitia were eliminated by a scalpel. Sterilization was done by immersing tissues into 70% ethanol for 5 min. After sterilization all steps were performed under sterilized condition at 4 °C. The tissues were washed in DI water to eliminate the residue of ethanol. The sterilized vessels were immersed into a 1.2% NaCl (Merck) solution for 2 h and in DI water for another 2 h. Then the samples were immersed into 0.025% trypsin (Gibco) solution with a gentle agitation for 24 h. This step was performed to eliminate the residual cell membranes in the tissue. Finally, 1% Triton X-100 (Sigma-Aldrich) was employed with a gentle agitation for 48 h to remove nonpolar molecules. The samples thoroughly were washed by DI water under agitation for 72 h to remove the residue of Triton X-100 (at 12 h intervals the water in the container was changed). Ultimately, the samples were immersed into a 1 × PBS (Sigma-Aldrich) solution containing 10% Pen-Strep for 3 days at 4 °C (the solution was replaced daily).

### Smooth muscle cell expansion

A10 cell line (NCBI Code: C600) was provided from National cell bank of Iran (NCBI) and was cultured in Dulbecco’s Modified Eagle Medium/Nutrient Mixture F-12 (DMEM/F12 GlutaMAX, Gibco) with 10% fetal bovine serum (FBS, Gibco) at 37 °C, 90% humidity and 5% CO_2_.

### Bioreactor for dynamic culture

In our previous work [30], a bioreactor was designed to simultaneously exert shear and tensile loadings to the tissue and simulate mechanical situation which the blood flow created for vessels. In this bioreactor, frequency and amplitude of both stresses are variable. Briefly, the cell-seeded scaffold was fixed at the bottom of the bioreactor chamber and filled by the culture medium. After starting of the bioreactor, rotation of the cone shape stirrer make a shearing regime on the scaffold surface. While the mobile grippers of the bioreactor applied cyclic stretches to the scaffold. After cell adhesion assay and obtaining the optimized mechanical stresses, the shear stress and the tensile strain for this research adjusted in 5 dyne/cm^2^ and 10% strain, respectively. Frequency of cycles set on 1 Hz to mimic heart rate.

### Histological analysis

Formaldehyde (4 wt%)-fixed samples were dehydrated and embedded into paraffin, then sectioned in 4 μm with a microtome. Hematoxylin and eosin (H&E) and Masson’s trichrome staining were employed according to the protocols provided by manufacturer. The stained slides were visualized with a microscope (Olympus, SZX 9, Tokyo, Japan).

### Scanning electron microscopy

After 1st and 3rd runs of culture in the bioreactor [30] under cyclic shear and tensile loadings. The cells-BS assemble were fixed with 2.5% glutaraldehyde overnight at 4 °C, and dehydrated in a series of graded ethanol (30, 50, 75 and 95%). After gold sputtering, the fixed samples were observed with a scanning electron microscope (SEM, FEI Quanta 450, Beaverton, OR, USA).

### Cellular compatibility of the BS

The BS effect on viability and metabolic activity of SMC were determined using 3-(4,5-Dimethyl-2-thiazolyl)-2,5-diphenyl-2H-tetrazolium bromide (MTT, Sigma-Aldrich) assay. The obtained BS was punched into a 96-well plate. SMCs were cultured in F12 with 10% FBS at 37 °C in a humidified atmosphere of 5% CO_2_. After reaching to 90% of confluency, they were trypsinized and suspended in complete medium with a density about 1 × 10^5^ cell/ml. Then, 50 μl of the suspension was dropped exactly on the center of each scaffold and left for 4 h. Later, the complete medium was gently added to each well and the samples were incubated for 24 h. After 24, 48, and 72 h, MTT solution was added to reach the final concentration equal to 0.5 mg/ml then the solutions were incubated for 4 h. The formed formazan crystals was dissolved by 100 μl dimethyl sulfoxide (DMSO, Sigma-Aldrich) under shaking for 3 h. The absorbance of the obtained supernatant was read at 570 nm using a plate reader (Bio-Tek instrument).

### Swelling ratio

Native blood vessel and BS were soaked in PBS for overnight and weighed. Then the samples were dried thoroughly at 50 °C, and swelling ratio was calculated based on the equation below:

$${\rm{SR}} = \frac{{W_w - W_d}}{{W_d}}$$

where SR, *W*_*w*_ and *W*_*d*_ are swelling ratio, the samples’ wet and dry weights, respectively.

### Opening angle

To evaluate the residual stress in the blood vessel before and after decellularization, vessel rings with same length were cut and their opening angles were measured (*n* = 7). This test repeated after recellularizing of the BS to reveal the role of cells in the residual stress.

### Cell adhesion assay

Cells were seeded on the BS, and the Polydimethylsiloxane (PDMS) membrane as control. In one series of samples, FBS was used to modify surface before seeding cells. After 24 h of incubation, the samples were placed into the bioreactor under shear stress of 2.5, 5 and 10 dyn/cm^2^, and tensile strain of 10 and 15%. The amount of attached cells was counted before and after exerting any force.

### Tensile test

The mechanical properties of natural vessel and BS were compared by uniaxial tensile test in the longitudinal and radial axes (*n* = 5) according to ISO 37:2005 [31]. All PBS-wetted samples with radius of 4.5 ± 0.3 mm and length of 30 mm were fixed to the universal testing machine (Hounsfield-H10KS, USA) and tensile tests were performed at rate of 10 mm/min in room temperature until samples rupture. The stress–strain curves were plotted and mechanical properties including breaking strength, elongation, and Young’s modulus were calculated by below equations:

$$E = \frac{{FL_0}}{{A_0\Delta L}}$$

$${\rm{Breaking}}\,{\rm{Strength}} = \frac{{F_M}}{{A_0}}$$

$${\rm{Elongation}}\,{\rm{at}}\,{\rm{break}}\,\left( {{{\mathrm{\% }}}} \right) = \frac{{\Delta L_B}}{{L_0}}$$

where *E* is Young’s modulus, *F* is the exerted force, *L*_0_ and *A*_0_ are the original length and cross-sectional area of the sample, *F*_*M*_ is the maximum force recorded during the tensile test, ∆*L* and ∆*L*_*B*_ are the sample length change and length change at breaking point, respectively. Due to two linear region in stress–strain curve, two Young’s moduli have been reported. The first region (in low strains) was mentioned as elastin Young’s modulus (*E*_e_) and the second region (in high strains) as collagen Young’s modulus (*E*_c_).

### Statistical analysis

Data statistical analysis and multiple comparisons between the groups was performed by one way ANOVA method and results are reported as the mean ± SD. *p* < 0.05 is considered to be significant.

## Results

**Fig. 1:**
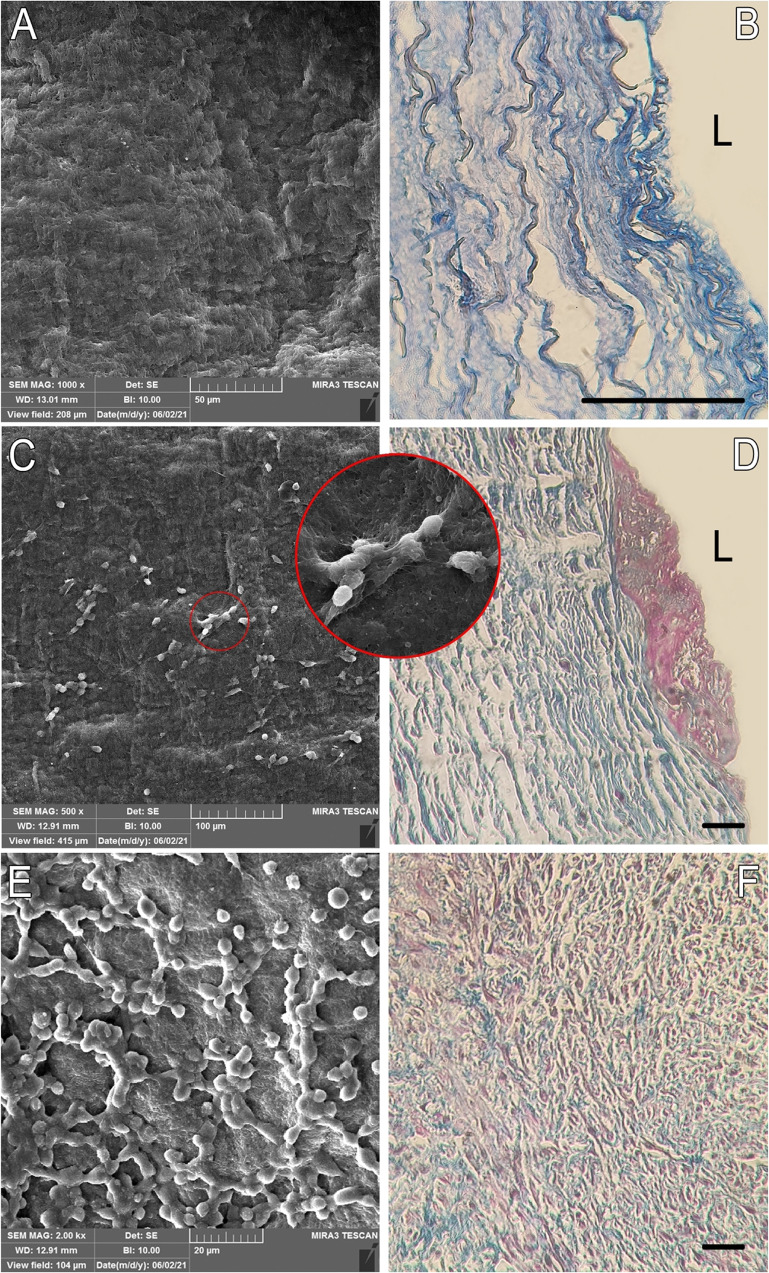
SEM images of biological scaffold before (**A**) and after 1 and 7 day cultivation (**C**, **E**) on the luminal surface which reveals cell proliferation and quality of the cell attachment on the surface of scaffold. Red circle in image **C** shows the start of colony formation. Transverse section of the same samples stained by the Masson’s trichrome (**B**, **D**, **F**), scale bar = 100 μm. The collagen filaments and cells are represented by blue and purple colors. In **D**, there is an agglomeration of cells on the lumen (L) which after 7-day they penetrated to the pores between collagen strings (**F**)

Figure 1A, C, E represents SEM images of longitudinal section of the acellular scaffold, before any cell culture, and also the ones cultured with SMCs for 1st, and 7th days. It is vital for a scaffold to have adequate sites for cell attachment then their proliferation is tuned. The form of cells, in points of adhesion to the acellular network and developing pseudopodia reveals that the microenvironment of the prepared BS is suitable for this aim. Moreover, according to Fig. 1B, D, F the Masson’s trichrome staining of transverse section of the same samples showed a high level of decellularization in the primary acellular scaffold and only the ECM has been remaining considering the population of the blue filaments. Upon recellularization, the attachment and proliferation of the cells on the lumen (L) area was clearly evident (purple color in Fig. 1D). After 7 days of cultivation, in addition to more proliferation, the cells migrated and infiltrated throughout the scaffold (Fig. 1F), and it was in correlation with SEM micrographs from the interface of the scaffold. The porous ultrastructure of the scaffold inherited from the native tissue and comprising interconnected macro and meso-porosities, accelerates cell migration, the transportation of the nutrients and cellular waste removal, and angiogenesis as well; these all are vital for neotissue formation.

**Fig. 2:**
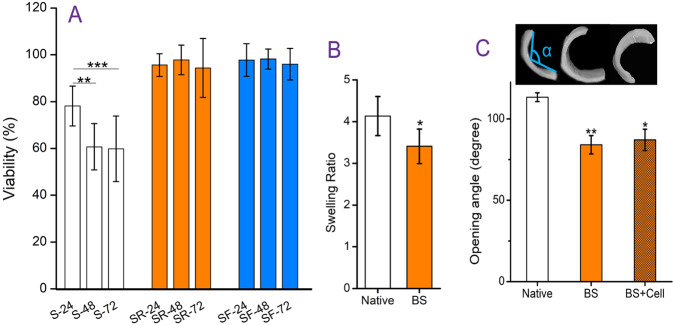
**A** Cell viability of SMC seeded on the acellular scaffold without (S) and with (SR) the rewashing step, and the acellular scaffold froze at −20 °C (SF) at 24, 48 and 72 h. MTT assay was utilized to evaluate cytotoxicity of the scaffold. The obtained data reveals in the S viability reduces significantly in each three-time points especially 48 and 72 h. However, in SR and SF viability reduction is much less than S. **B** Swelling ratio test. The native and BS samples were weighed in wet and dry conditions, and swelling ratio was calculated. **C** Measurement of the residual stress by opening angle test. The Native sample which is a fresh coronary artery tissue without any process. The BS sample is biological scaffold obtained from the same tissue. The BS + Cell sample is the BS after 3-day SMC cultivation. *p < 0.01, **p < 0.001, ***p < 0.0001

Figure 2A shows the results of MTT assay. The S sample is the BS after 1 month storing in fridge at 4 °C, and the SR is the S sample after rewashing in F12—10% FBS for 3 days. Long-term storage in fridge initiates the degradation of ECM and further pH changes that affects cell attachment, and consequently cell viability. After 48 and 72 h, cell apoptosis caused a more toxic microenvironment. However, in case of SR, the pH of BS recovered due to washing and reached to a suitable condition for the cells, therefore the viability percentage around 100% till 48 h was met. After 72 h, the viability of cells was fallen, presumably because of and excessive consumption of the nutrients available in the medium. Herein, one can deduce that BS has a positive impact on the kinetics of cell proliferation. For BSs stored at −20 °C, without any rewashing step, no remarkable cytotoxicity was observed. Figure 2B represents the comparison between swelling ratio of the native and the decellularized vessels. Upon removing the cells from ECM, the swelling ratio decreased significantly. Figure 2C shows the opening angle of the native, BS and the cell-seeded BS as well. The native vessels had the highest amount of opening angle (about 113.3°), indicating the storage of the highest amount residual stress in the native tissue. After decellularization, the residual stress declined significantly and reached to about 84.1°. However, upon recellularization by SMCs (after 3 days of culture) the angle increased and reached about 87°. These findings suggest that filling the spaces available in the protein network of the ECM with cells significantly impacts the amount of residual stress in blood vessels. Considering the structure of a blood vessel as a multilayer cylinder, opening angle is highly associated to the ratio of the pressure of inner layer over the outer one.

**Fig. 3:**
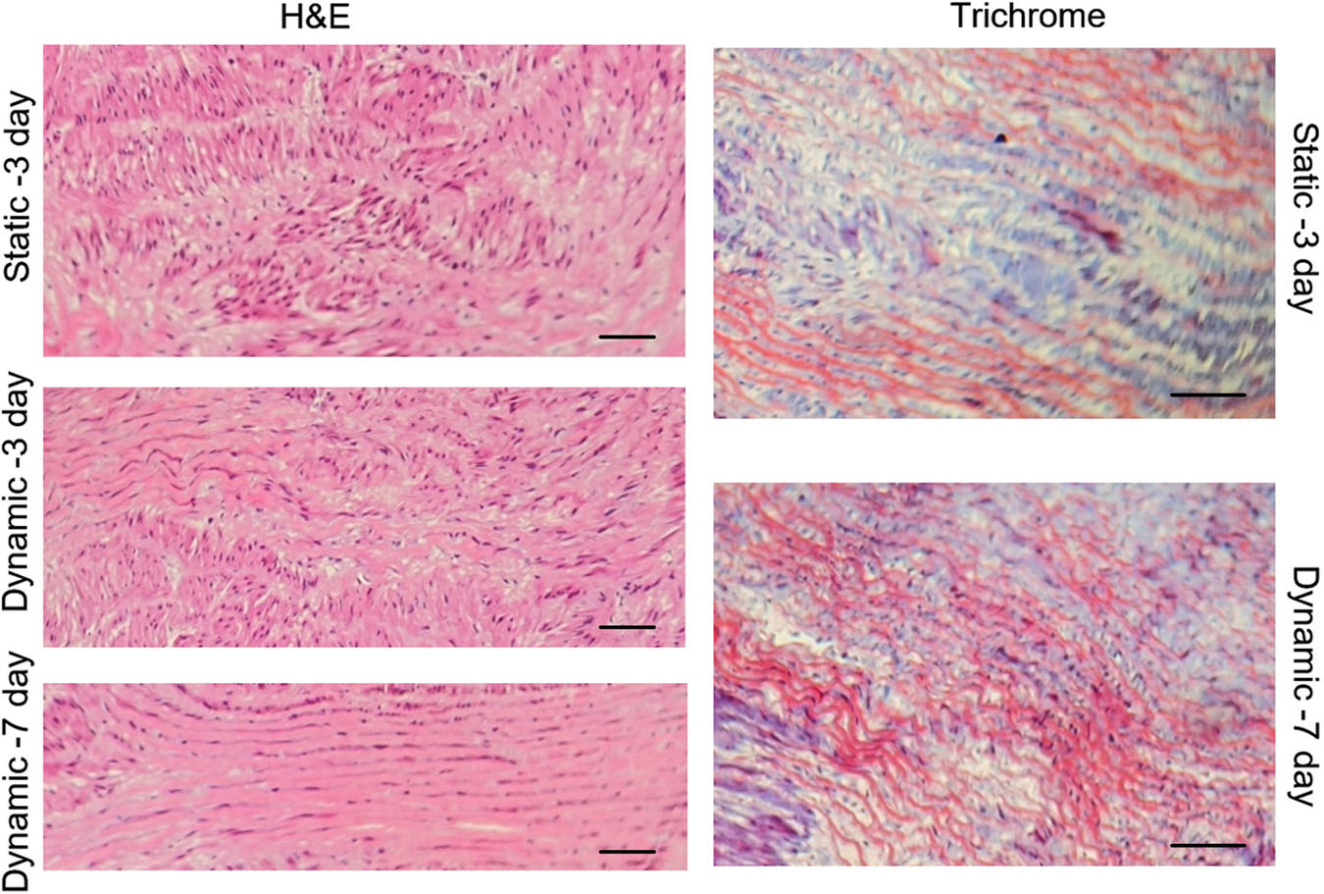
Histological images of the biological scaffold after 3-day static, 3-day dynamic and 7-day dynamic SMC cultivation with the H&E and Masson’s trichrome staining. Dynamic culture made collagen filaments aligned and compressed, and helped cells penetration (scale bar = 300 μm)

Figure 3 shows the histological sections of the BS after recellularization under static and dynamic conditions. The H&E images revealed remarkable penetration of the cells into scaffold structure. In the dynamics condition due to shear stress (5 dyne/cm^2^) and strain (10%) exerted to the scaffold, the compression of structure can be observed, and thickness of scaffold reduced. On the other hand, collagen filaments become aligned and parallel with each other which is the natural structure of blood vessels.

**Fig. 4:**
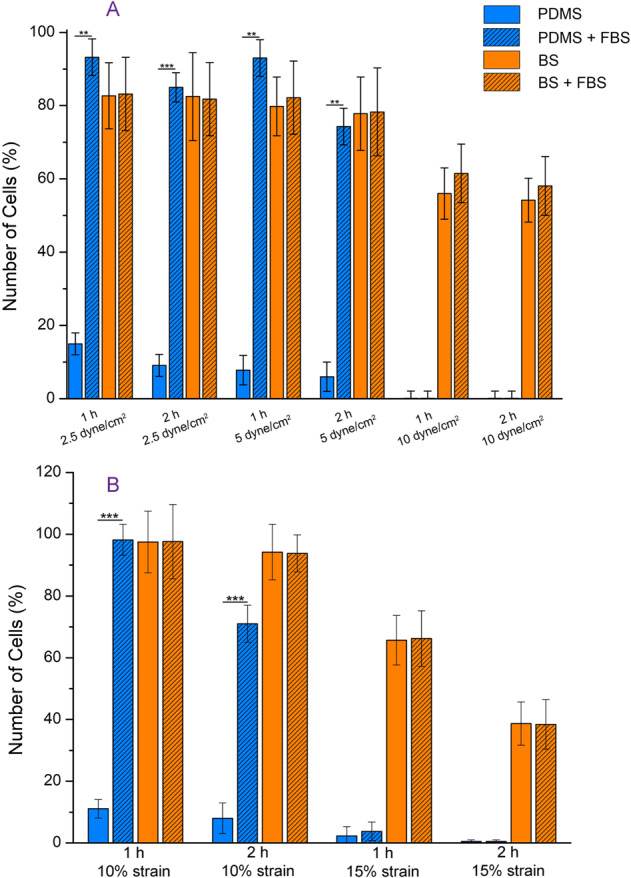
Cell adhesion assay. Percent of remaining cells on the scaffold after exerting cyclic shear stress (**A**) and stretch (**B**) to the scaffold were calculated. The blue column is PDMS and the orange ones BS. FBS treatment was used to improve cell adhesion. **p < 0.01, ***p < 0.001

Figure 4 represents cell adhesion strength to the acellular BS. PDMS was employed as a control, and FBS was consumed to improve cell adhesion to the surface. Three amount of shear stresses (2.5, 5 and 10 dyne/cm^2^) were exerted to the both scaffolds with and without FBS treatment for 1 and 2 h. As shown, the proteins present in FBS significantly promoted PDMS surface; however, changes on the BS is negligible due to its natural composition consisting of several kind of proteins available in ECM. For shear stresses of 2.5 and 5 dyne/cm^2^, PDMS showed acceptable results but 10 dyne/cm^2^ pushed out all the cells. However, the BS represented more steady performance with average of 80% which in shear stress of 10 dyne/cm^2^, it decreased to around 60%. Moreover, time has more negative effects on the PDMS scaffold rather than the BS. For cyclic stretch, the same behavior can be observed. In 10% strain, the FBS modified PDMS showed same performance with the BS at 1 h but drooped at 2 h. In 15% strain, the BS had an obvious reduction; however, the PDMS lost all its cells.

**Fig. 5:**
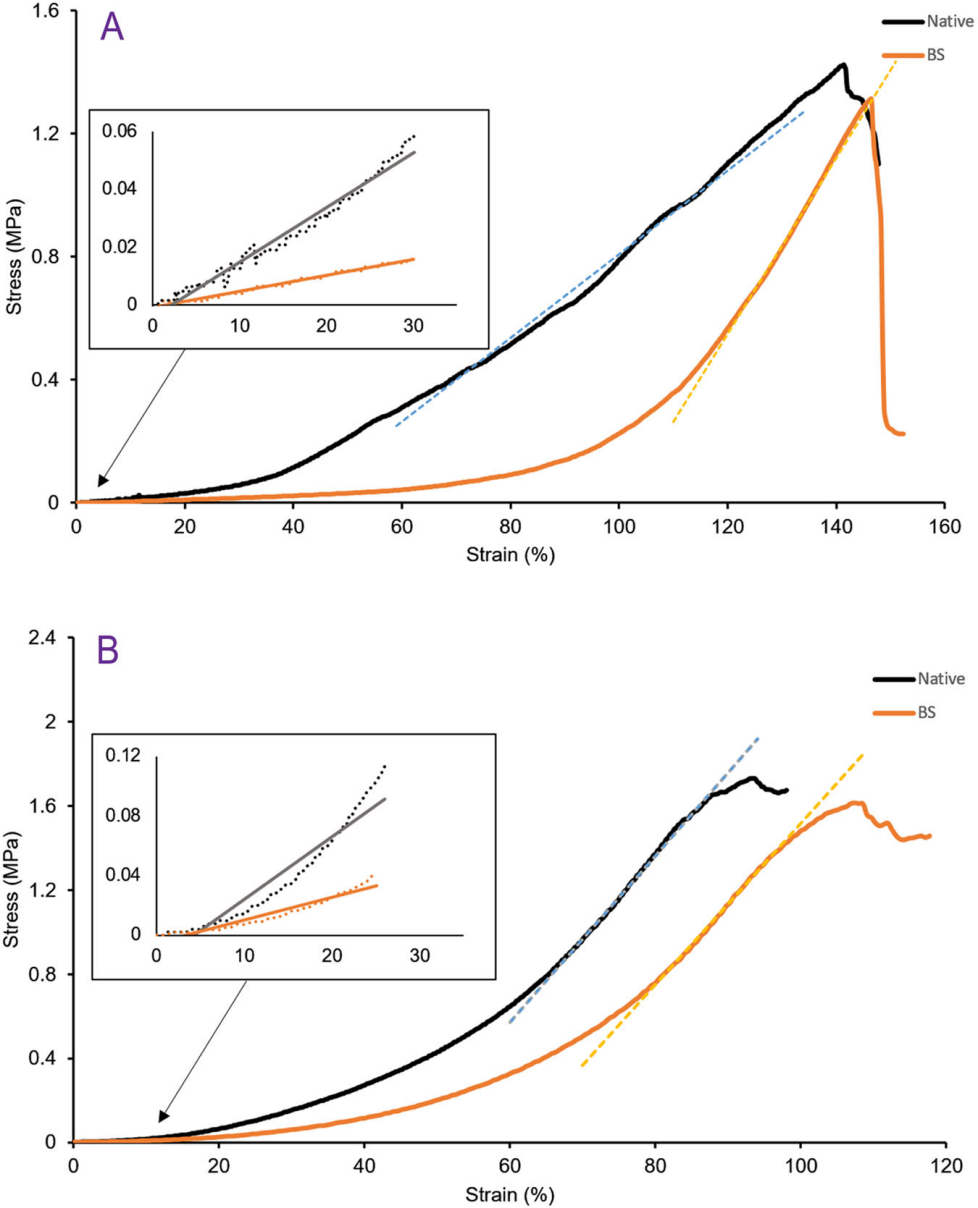
Tensile test. Stress–strain curve of native blood vessel and BS in longitudinal (**A**) and radial (**B**) axis. Inlet graphs show stress–strain curve in the small strains with more details which belongs to elastin region. Linear part in high strains is collagen region

Figure 5 shows the stress–strain curves for the native ovine coronary artery and the decellularized scaffold. Each curve represents three regions. The first linear region in small strains is associated with the elastin fibers of the tissue. The small forces of this region cannot rearrange collagen filaments, and slope of this region gives the elastin Young’s modulus. As shown, detaching the cells from ECM had negative effect on elastin net. The second nonlinear region is transition region that upon increasing the stress the collagen filaments in the ECM structure start sliding over each other and aligning with the direction of the applied stress. It is obvious in Fig. 5 in both longitudinal and radial direction, eliminating cells as anchorage points between these filaments made them more flexible and they slid with lower forces and got more stretched. The interesting point is blood vessel tissue reveal anisotropic behavior, and in the longitudinal direction the transition region is very short and negligible. The final linear region is collagen region which happens in high strain, and stress exerted to aligned collagen filaments. The slope of this region shows the collagen Young’s modulus. This region end at the ultimate tensile strength (UTS) by starting rupture in the filaments. As the data represents this decellularization process didn’t affect harshly on the ECM collagen quality.

In Table 1, mechanical properties of the native blood vessel and BS including elastin (*E*_e_) and collagen (*E*_c_) regions Young’s modulus, UTS and elongation at break point were compared (*n* = 5). As mentioned, elastin and collagen are main structural proteins in the ECM, and investigation of their behavior can provide a clear vision about ECM mechanical behavior.

**Table 1:** Mechanical properties of native blood vessel and biological scaffold

Stress mode	Sample	*E*_e_ (MPa)	*E*_c_ (MPa)	UTS (MPa)	Elongation at break (%)
Longitudinal	Native	0.20 ± 0.02	1.36 ± 0.1	1.4 ± 0.05	146 ± 5
	BS	0.06 ± 0.01	2.87 ± 0.2	1.3 ± 0.04	155 ± 10
Radial	Native	0.43 ± 0.04	3.96 ± 0.1	1.7 ± 0.04	98 ± 10
	BS	0.17 ± 0.03	3.83 ± 0.3	1.6 ± 0.08	117 ± 10

## Discussion

For decades, researchers have been investing lots of time and effort to develop suitable scaffolds for blood vessel tissue engineering. However, the delicate and complex multilayer structure and continuous cyclic stresses made it challenging, especially in points of small arteries. A variety of synthetic and natural polymers with different fabrication methods have been tried so far, but they are still lacking an appropriate chemical condition or ultrastructure, so are usually unable to provide neither the signaling pathways nor the microenvironment appropriate for cell attachment and proliferation [32, 33].

Decellularized scaffolds gained a lot of interests recently in light of their natural microstructure composed of macro and mesoporosities, and abundant cytokines and growth factors that could be preserved within the context of the scaffold, however, both are contingent upon the processing. There are two main approaches to decellularize a tissue; chemical and physical methods. Within chemical approaches detergents, chelating agents, acid/bases, enzymes, etc., are utilized commonly and all alternate the native ultrastructure somehow, but physical methods usually have a minimal impact on the structure and yield almost an intact ECM, though do not have the functionality of chemical methods in terms of removing cellular components [12–22].

In this study, by combining physical and chemical methods, we developed a novel decellularization protocol with reduced concentration of chemical reagents and able to have a minimal impact on native ECM structure. Upon exerting cyclic osmotic pressures on the tissue, the majority of cellular wastes were eliminated without any detrimental damage to ECM proteins. Also, keeping the temperature of whole decellularization procedure at 4 °C lowered the kinetic rate of the reactions and decreased the probability of structure deterioration; these all were reflected in mechanical examinations. In fact, the established method eliminated cells thoroughly without any fundamental rupture or resorption of the collagenous strands endowing a suitable microstructure and porosity distribution facilitating cell infiltration, migration, and attachments. SEM images demonstrated developing pseudopodia and the proliferation of SMCs on the acellular scaffold (Fig. 1).

Though, based on a MTT assay the BS stored at −20 °C was found cytocompatible, the ones stored at 4 °C for a month were detrimental to cells and the percentage of cell viability decreased, significantly, therefore it is concluded that the storage condition is impactful on BS properties. The swelling ratio of the BS was found lesser than the native vessel, thus lower water absorption upon immersion into an aquatic microenvironment helped the acellular structure to properly maintain the natural integrity. In accordance with the results of opening angle of the obtained BS, the residual stress dropped significantly as well. However, upon recellularizing and culture with SMCs, BS recovered the opening angle. It is deduced that the presence of cells significantly impacts the residual stress within the structure upon filling the concentric layers of the ECM (Fig. 2).

In a blood vessel, cells act as junction points and connect the collagen fibers. After decellularization these fibers become loosened and their distance is increased. The culture into bioreactor [30] significantly improved cell migration and made the fibers tighter. After 7 days of dynamic culture, compact parallel fibers with smooth distribution of cells between them were obtained which resembled to the natural structure of the tissue (Fig. 3). Moreover, the bioreactor with adjustable cyclic tensile and shear stresses simulated the mechanical condition of blood flow in vessels. In this study, we employed various amount of stresses to quantify the cellular adhesion to the scaffold. As mentioned, PDMS scaffold as an example of synthetic scaffold needs surface modification with some proteins to anchor the cells. However, BS is made of natural structural polypeptides, cytokines, and growth factors do not require any surface modification, and treatment with FBS did not have any remarkable effect on its functioning in terms of cell attachment and proliferation. In low tensile and shear stresses, both of the PDMS and the BS showed satisfactory performance. But in higher stresses, cells easily detached from the PDMS scaffold. The other important characteristics of a scaffold are durability referring to the stability of the scaffold once implanted and the probability of failure (Fig. 4).

Having a tensile strength similar to natural vessels and an elongation comparable with or even more than natural one showed that the collagenous fibers of ECM remain intact after decellularization. However, changes in delicate proteins e.g., elastin is more probable. Removing cells from vessel tissue has an impact on transition region of stress–strain curve. Because after decellularizing, collagenous fibers could move more freely and slide on each other, lower forces are required for the transition region. On the other hand, when these fibers are not confined to the cell adhesion molecules can have more elongation [34]. Furthermore, native blood vessel and the BS were comparable within the final linear region of the curve, the slope, and the UTS. All revealing the presence of cells do not have any impact on the Young’s modulus, and the UTS as well. Structural proteins of ECM, mainly collagen, are responsible for it and when their amounts are comparable it means these structural proteins did not receive any damage during the decellularization process. It is vital for a vascular implant to have similar mechanical properties with the host vessel, and to have so higher or lower strength and stiffness both causes problems in long-term (Fig. 5). Interestingly, blood vessels showed orthotropic behavior in longitudinal and radial directions mainly because of collagen fibers direction [14]. As shown in Fig. 5, the nonlinear transition region in longitudinal direction was shorter than radial direction. The decellularization extended this transition region in the longitudinal samples. It can be realized that the cells confine collagenous fibers narrower in this direction.

## Conclusion

Briefly, we developed a chemiphysical method for the decellularization of coronary arteries resulting to obtain a biological scaffold with intact ultrastructure which can be used for tissue engineering of blood vessel without further modification. In comparison to the scaffold with artificial materials, this type of acellular scaffold is the closest one to the natural microenvironment, and is able to satisfy both of mechanical and cellular properties. Studying mechanical behavior of acellular and native blood vessel tissues revealed interesting function of cells in the transition region of the stress–strain curve and the residual stress of the tissue.
